# *Lilium pseudonanum* (Liliaceae), a Rare and Cryptic Species From Southeast Xizang, China

**DOI:** 10.1002/ece3.71738

**Published:** 2025-07-10

**Authors:** Xiaojuan Chen, Yumei Yuan, Yundong Gao

**Affiliations:** ^1^ Mountain Ecological Restoration and Biodiversity Conservation Key Laboratory of Sichuan Province, Chengdu Institute of Biology Chinese Academy of Sciences Chengdu China; ^2^ China‐Croatia Belt and Road Joint Laboratory on Biodiversity and Ecosystem Services, Chengdu Institute of Biology Chinese Academy of Sciences Chengdu China; ^3^ University of Chinese Academy of Sciences Beijing P.R. China

**Keywords:** conservation, cryptic species, hybrid origin, *Lilium nanum*, *Lilium pseudonanum*

## Abstract

*Lilium pseudonanum*, a new species endemic to highly restricted habitats in the eastern Himalayas, has been identified and taxonomically distinguished from the morphologically similar *L. nanum* . Genetic analyses utilizing nuclear and chloroplast datasets have conclusively established *L. pseudonanum* as a distinct species, revealing a complex relationship with *L. nanum* and other related taxa, despite the historical classification of the former two as conspecific. Genomic evidence suggests a potential hybrid origin for *L. pseudonanum.* Nuclear‐plastid discordance indicates chloroplast capture from the Duchartrei clade and nuclear introgression from the Lophophorum clade. Furthermore, ADMIXTURE analyses reveal a tripartite ancestry, while TreeMix analysis has detected bidirectional gene flow from both putative parental lineages. In contrast, morphological principal component analysis (PCA) has shown no significant differences between the two species, except for the notably larger basal leaf blades in *L. pseudonanum*, highlighting its cryptic nature. Ecologically, *L. pseudonanum* occupies habitats characterized by higher summer precipitation and prolonged snow accumulation during winter, providing greater moisture availability compared to the drier habitats of *L. nanum.* This suggests a degree of ecological niche divergence between the two species. Environmental niche modeling (ENM) predicts that the distribution of *L. nanum* may shift in response to global warming, potentially driving its upward migration to higher elevations or latitudes. This northward or altitudinal movement could lead to overlapping habitats with *L. pseudonanum,* raising critical concerns about habitat loss and the risk of genetic introgression. Such introgression could threaten the taxonomic distinctiveness and ecological stability of *L. pseudonanum*. Given its extremely restricted geographic range and small population size, *L. pseudonanum* has been classified as “Critically Endangered” (CR). As a result, urgent actions are needed to confirm its taxonomic status and implement comprehensive conservation measures to ensure its survival.

## Graphical abstract

*Lilium pseudonanum* is a newly identified, critically endangered species endemic to the eastern Himalayas, distinguished from *L. nanum* through genomic evidence despite its morphological similarity. Genetic analyses reveal a complex hybrid origin involving chloroplast capture and nuclear introgression, while ecological niche modeling predicts future habitat overlap due to climate change, raising conservation concerns. Given its restricted range and small population, urgent measures are needed to protect this cryptic species from genetic introgression and habitat loss.

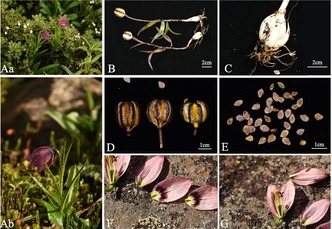

## 1 Introduction

Lilies, in a general sense, refer to plants belonging to the genus *Lilium* within the monocotyledonous family Liliaceae (Linnaeus 1753; Liang and Tamura 2000). A total of 118 documented species of this genus are distributed worldwide, with a primary concentration in temperate and cold regions of the Northern Hemisphere (POWO 2024; Liang 1995; Liang and Tamura 2000). China hosts the highest diversity of *Lilium* species and is recognized as the global center of origin for the genus (Gao, Harris, et al. 2013; Givnish et al. 2020). Notably, the region spanning the Qinghai‐Tibet Plateau (QTP) and the Hengduan Mountains serves as a diversity hotspot, containing numerous alpine *Lilium* species, particularly those in section *Lophophora* (Bureau & Franch.) Wang et Tang, exemplified by *Lilium lophophorum* (Bur. et Franch.) Franch. (Watanabe et al. 2021). This section encompasses more than eight species, collectively termed clade Lophophorum (*sensu* Gao, Harris, et al. 2013), distributed across the Himalayas and adjacent Hengduan Mountains in southwestern China and neighboring regions (Gao, Harris, et al. 2013; Givnish et al. 2020).

Taxonomically, *Lilium* species have been predominantly classified based on floral characteristics, both at the section and species levels (Comber 1949; Baranova 1988). However, this method often complicates species identification due to prevalent parallel evolution within the genus (Gao et al. 2015; Huang et al. 2023; Yuan and Gao 2024; Feng et al. 2025; Duan et al. 2025). For example, *Lilium yapingense* Y.D. Gao et X.J. He was initially considered conspecific with *Lilium nanum* Klotzsch because both species exhibit purple, campanulate, pendulous flowers, linear leaves, and a dwarf growth habit (Gao, Zhou, and He 2013). However, detailed examinations of key morphological features, such as nectar furrows, soon highlighted their distinctions. This demonstrates the value of a meticulous morphological approach when an extensive range of traits is considered. Such superficial resemblances must be carefully disregarded when determining species status. In many instances, however, morphology alone proves inadequate for distinguishing distantly related yet morphologically similar species (DeSalle et al. 2005; Bickford et al. 2007; Struck et al. 2018). Particularly, cryptic species pose significant challenges as they exhibit high genetic divergence despite their morphological resemblance (Bickford et al. 2007). The morphological similarity among species often fails to reflect their true evolutionary relationships within *Lilium* (Gao et al. 2015; Yuan and Gao 2024; Feng et al. 2025; Duan et al. 2025). Since species divergence does not always coincide with notable morphological differentiation, cryptic species are generally rarer than species discernible solely by morphology (Bickford et al. 2007). Consequently, cryptic taxa are often overlooked and misclassified as a single nominal species by taxonomists (Knowlton 1993). Detailed phylogenetic analyses are crucial for accurately identifying such genetically distinct lineages (Li, Xiang, et al. 2020; Borges et al. 2022). Moreover, under similar selective pressures, distantly related species may develop convergent phenotypes, while genetically close species may display morphological divergence to adapt to different ecological niches or competitive environments (Gao et al. 2015; Yuan and Gao 2024; Duan et al. 2025; Givnish et al. 2020).

Although phylogenetic analyses can identify cryptic species and elucidate their hidden status, challenges remain, primarily related to discordances between morphology and molecular data. Such discrepancies are often attributed to incomplete lineage sorting (ILS) or introgression. Distinguishing between these phenomena is critical for accurate taxonomic resolution. ILS is typically seen as an issue complicating phylogenies, while introgressive hybridization significantly influences intraspecific diversity and creates hotspots of genetic variation (Hewitt 2011; Marques et al. 2018). Hybridization acts as a fundamental ecological process, shaping biodiversity patterns and impacting genetic structures and morphological characteristics of species (Canestrelli et al. 2010). Furthermore, hybrid species, which are frequently overlooked due to their morphological similarity arising from genetic affinity, may represent distinct evolutionary lineages with unique genetic resources. Consequently, they hold significant conservation importance (Pace 2021).

To distinguish ILS from hybridization, well‐resolved phylogenies based on comprehensive sampling and appropriate methodologies are required. The integration of molecular tools with diverse approaches has proven effective in identifying hybrid zones and uncovering overlooked cryptic species (Barton and Hewitt 1985; Dépraz et al. 2009; Lemmon et al. 2007; Kauserud et al. 2007). Recent field surveys conducted by the authors in southwestern China, focusing on the genus *Lilium*, have provided valuable new specimens of dwarf lilies with bell‐shaped flowers belonging to section *Lophophora*. These samples offer a critical opportunity to further evaluate species diversity and explore the evolutionary history within this alpine group. Comprehensive sampling and subsequent analyses revealed a previously unrecognized species that had long been misclassified as *L. nanum* due to its high morphological resemblance (Figure S1). This discovery strongly suggests the presence of an overlooked cryptic species.

In the present study, a multidisciplinary approach is applied, integrating molecular, morphological, and ecological datasets, to resolve the taxonomic status of this putative cryptic species and investigate its potential origin and ecological niche. The objectives of this work include: (1) verifying the taxonomic status of the suspected novel species; (2) identifying diagnostic morphological characteristics, if present, that separate the new taxon from its closest relatives; (3) elucidating the origin and cryptic nature of the species; and (4) conducting ecological niche comparisons and predictive modeling under climate change scenarios to assess conservation priorities. These findings are expected to provide deeper insights into the taxonomy, evolution, and conservation of *Lilium* species, particularly within the unique environmental contexts of the Qinghai–Tibet Plateau and Hengduan Mountains.

## 2 Materials and Methods

### 2.1 Sample Collection and Field Observation

**FIGURE 1:**
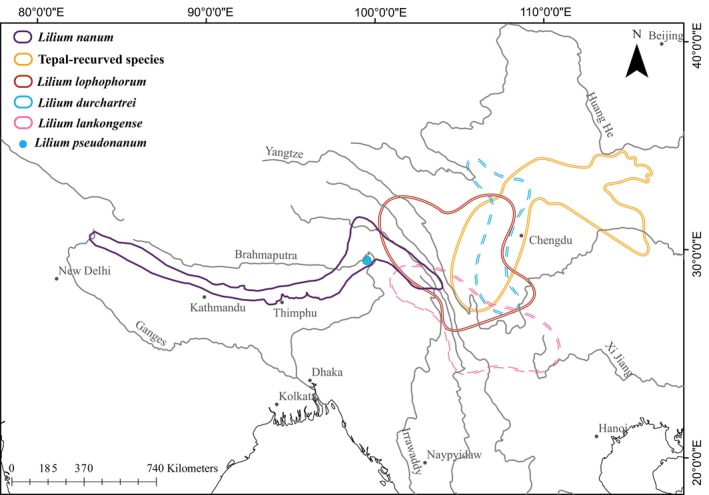
Distribution range of *L. pseudonanum* and related species, including tepal‐reflexed Lophophorum species such as *L. fargesii*, *L. matangense*, and *L. stewartianum* (for details, refer to the main text).

The study materials were collected during 2021–2024 from multiple sites in southwestern China. The putative new species, in particular, was collected in 2022 from Linzhi City, Xizang, China (the precise location is withheld for protection and conservation reasons). Leaves from multiple individuals were stored separately in either silica gel or liquid nitrogen prior to being transported to the −80°C refrigerator. The vouchers are currently preserved at the Herbarium of Chengdu Institute of Biology, Chinese Academy of Sciences (CDBI). To estimate the population size and evaluate the threat status of the species, the Extent of Occurrence (EOO) and Area of Occupancy (AOO) were calculated using the GeoCAT software (Bachman et al. 2011), based on field observations. The distribution range of *L. pseudonanum*, as determined from field surveys, is depicted in Figure 1.

### 2.2 Acquisition and Analysis of Morphological Trait Data

Initially, a preliminary comparison was conducted to assess the potential related species within the Lophophorum clade, providing an overview of the characteristics of the putative new species. Subsequently, a more detailed comparison was performed with the most closely related species to refine the analysis at a finer scale. This study collected morphological data from eight *Lilium pseudonanum* individuals through field measurements and compared them with specimens from 14 populations (47 individuals) of *L. nanum* available in the Chinese Virtual Herbarium (https://www.cvh.ac.cn/). Thirteen quantitative traits were selected to construct a morphological matrix, including plant height, basal leaf length, basal leaf width, basal leaf length/width ratio, central leaf length, central leaf width, central leaf length/width ratio, apical leaf length, apical leaf width, apical leaf length/width ratio, fruit length, fruit width, and fruit area. Leaf length was measured from the leaf base to the leaf tip, averaging 5–10 leaves; leaf width was measured at the widest part of each leaf, averaging 5–10 leaves. Flower stem length was measured from 5 to 10 individuals, and the average value was taken as the stem length. Fruit length was measured from the top of the flower stem to the top of the ovary tube, and the average value from 5 to 10 individuals was recorded as the ovary tube length (Figure S2).

To optimize the utility of the morphological data, this study employed a multivariate normal mixture model‐based morphometric approach to assess putative interspecific morphological discontinuities (Zapata and Jiménez 2012). Initially, principal component analysis (PCA) was conducted to reduce the dimensionality of continuous morphological traits, focusing on the first two principal components to construct a multidimensional morphospace. Subsequently, saddle‐line manifold theory (Ray and Lindsay 2005) was applied to evaluate multimodality in probability density functions along the interspecific mean vector, providing statistical validation for the existence of morphological gaps.

All computations were performed using R version 4.3.1. Parameters of the multivariate normal mixtures were estimated with the mvtnorm package (Genz et al. 2009), while elliptical tolerance regions at the 95% confidence level were generated using the ellipse package (Murdoch and Chow 2007) to quantify β‐overlap proportions between species‐specific morphometric distributions (Mathew 1999). A prior frequency threshold of 0.1 was established, wherein interspecific overlap proportions falling below this criterion provided statistical support for species delimitation. This methodology quantitatively assesses the distributional topology within morphospace, establishing probabilistic criteria for taxonomic boundaries while circumventing the strong hierarchical assumptions that characterize conventional cluster analyses.

### 2.3 Molecular Property and Phylogeny Inference

Genomic DNA was extracted from silica gel‐dried leaves using a modified cetyltrimethylammonium bromide (CTAB) method (Allen et al. 2006). Paired‐end sequencing libraries were then constructed with insert sizes of approximately 350 bp, followed by sequencing on the DNBSEQ‐T7 platform (Beijing Genomics Institute, BGI), with a depth of about 0.1~0.2 × (10G pair ending reads). About 13 Gb of raw data were filtered by fastp v0.23.2 (Chen et al. 2018). The Internal Transcribed Spacer (ITS1, 5.8S, and ITS2) and chloroplast genome of the new species were then assembled using GetOrganelle v1.7.6.1 (Jin et al. 2020) with default parameters.

The newly generated ITS and complete chloroplast sequences were deposited in GenBank (https://www.ncbi.nlm.nih.gov/). Molecular phylogenetic analysis was conducted using the maximum likelihood (ML) method and Bayesian inference (BI) with the PhyloSuite v1.2.2 (Zhang et al. 2020). The selection of 38 reference sequences from GenBank aimed to capture a representative sample of the diversity within the genus *Lilium*, specifically targeting species akin to the new species, such as members of the clade Lophophorum, as well as three distinct outgroups (*Fritillaria*, *Notholirion*, and *Cardiocrinum*) In PhyloSuite, Mafft (Katoh and Standley 2013) was used to perform multiple sequence alignment in the default alignment mode with the “‐‐auto” strategy. Gblocks (Talavera and Castresana 2007) was then applied to remove ambiguous sites and missing data. The aligned sequences were subsequently merged into a single alignment and converted into Nexus format files. The most appropriate evolutionary model was selected using ModelFinder (Kalyaanamoorthy et al. 2017).

Maximum likelihood (ML) analysis was performed using IQ‐TREE v2.0 (Minh et al. 2020). Bootstrap analysis with 1000 replicates was conducted to assess branch support. Bayesian phylogenetic (BI) analysis was performed using MrBayes (two parallel runs, 10,000,000 generations), with the initial 25% of sampled data discarded as burn‐in. The results indicated that the best‐fit model for ITS data under ML was TIMe, and the best‐fit model under BL was SYM; for chloroplast sequences, the best‐fit model under ML was GTR, and the best‐fit model under BI was TVM. Chloroplast CDS was also extracted to construct the phylogenetic tree using the same method as above, with the best‐fit model for both ML and BI being GTR. The phylogenetic trees were visualized using iTOL v6 (https://itol.embl.de, Letunic and Bork 2024).

### 2.4 Chloroplast Genome Synteny Analysis

The chloroplast genome of *L. pseudonanum* was annotated, and manual corrections were made using Geneious Prime v2023.1.2 (Biomatters Ltd., Auckland, New Zealand), based on the plastome of *L. lophophorum* (MK493298). Synteny analysis was performed using Geneious software, based on the Mauve multiple genome alignment method, comparing the chloroplast genomes of *L. pseudonanum* with those of *L. lankongense* Franch. (MK757466) and *L. duchartrei* Franch. (MN745200), obtained from the NCBI database.

### 2.5 Potential Habitat Suitability Zones of *L. nanum*

#### 2.5.1 Data Sources for Species Distribution and Study Area

Species distribution data were collected from field sampling by the research team, as well as such collection records with detailed locality. A total of 30 populations of *L. nanum* were sampled with coordinate data. Boundary data for the QTP were obtained from the National Qinghai‐Tibet Plateau Scientific Data Center (https://data.tpdc.ac.cn/). For the current and future bioclimatic conditions, the study utilizes open data provided by WorldClim V2.1 (https://worldclim.org/). Given China's unique geographical context, the research employs the BCC‐CSM2‐MR climate model from the Sixth Coupled Model Intercomparison Project (CMIP6), along with climate scenario data for the 2050s and 2070s under two Shared Socioeconomic Pathways (SSPs): SSP126 and SSP585.

#### 2.5.2 Analysis of Environmental Factor Correlation and Model Variable Selection

When constructing the MaxEnt model, high correlation among variables can lead to over‐fitting, and thus reduce the model's predictive ability. Therefore, it is necessary to assess whether there is any multicollinearity among variables. The MaxEnt model evaluates the contribution of all environmental variables to the suitable habitat distribution of *L. nanum*, removing variables with zero contribution. Pearson correlation coefficients were calculated for the initial selection of 19 bioclimatic variables (|*r*| > 0.8). Ultimately, seven environmental variables were selected for *L. nanum* modeling (Bio‐1, Bio‐2, Bio‐3, Bio‐12, Bio‐15, Bio‐18, Bio‐19).

This study employed the MaxEnt version 3.4.1 model (Phillips et al. 2006) to simulate the potential distribution of *L. nanum* across different temporal periods. A total of 30 occurrence points, along with climate variables relevant to each period, were incorporated into the model, with the climate variables treated as continuous data. To evaluate the contribution of each climate variable to the distribution of *L. nanum* , a Jackknife procedure was utilized to assess variable importance, which included the calculation of the contribution rate, normalized training gain, and response curves. The dataset was partitioned, allocating 25% of the species occurrence data for the testing set and 75% for the training set. The Bootstrap method was conducted for 10 repetitions, adhering to a maximum iteration limit of 5000. The output format was designated as Logistic, while all other parameters were maintained at their default settings. The average of the 10 simulation results was utilized to construct the potential distribution of *L. nanum* across the various periods. For the analysis, the natural breaks (Jenks) method was applied to categorize suitable habitats into four distinct levels.

### 2.6 Phylogenomic Network Inference of Paleohybridization

The preliminary results of the ITS and chloroplast genome phylogenies reveal discordances that may indicate the involvement of incomplete lineage sorting (ILS) or hybridization in the formation of the putative cryptic species. Consequently, a more detailed analysis utilizing transcriptome data was performed, incorporating a reduced sample size that includes representatives of related species identified through the initial phylogenetic analysis. Samples were sent to BGI for RNA extraction, library preparation, and sequencing.

The raw data underwent quality filtering using fastp v0.23.2 (Chen et al. 2018), followed by adapter trimming and PCR duplicate removal. A multitissue transcriptome assembly was conducted with Trinity v2.13.2 (Grabherr et al. 2011), with redundancy reduction achieved via CD‐HIT‐EST v4.8.1 (Li and Godzik 2006) at a 90% similarity threshold. Open reading frames (ORFs ≥ 100 amino acids) were identified using TransDecoder v5.5.0. Orthologous gene families were clustered from 47,431 protein sequences across 10 species using OrthoFinder v2.5.4 (Emms and Kelly 2019), resulting in 4214 low‐copy orthogroups (≤ 3 copies per species) designated as the core dataset.

Amino acid alignments were iteratively refined with MAFFT v7.505 (Katoh and Standley 2013), and codon‐level alignments were generated by mapping coding sequences via pal2nal v14 (Suyama et al. 2006). A species tree was reconstructed using ASTRAL v5.7.3 (Zhang et al. 2018) to coalesce the 4214 gene trees under a multilocus coalescent model. Additionally, GRAMPA v1.6 (Blischak et al. 2020) was employed for multilabel tree reconciliation, allowing for the quantification of discordance between gene trees and the species tree, and detecting topological conflicts at nodes with bootstrap support ≥ 80%, thereby inferring potential paleohybridization or introgression events.

### 2.7 Population Genomic Analyses

The exceptionally large genome size of *Lilium* species presents significant technical challenges for de novo genome assembly and population resequencing. This study innovatively utilizes a full‐length transcriptome as a reference framework for population genomic analysis. Experimental samples were collected from individuals of *Lilium regale* E.H. Wilson that were transplanted to our institutional garden, with leaf tissues and floral organs sampled at three developmental stages to encompass complete lifecycle transcriptomes.

RNA extraction, library preparation, and sequencing were performed on the PacBio Sequel platform by Beijing Novogene Bioinformatics Technology Co. Ltd., adhering to standardized protocols outlined by Li, Jiang, et al. (2020). Transcriptome alignment was conducted using BWA‐MEM v0.7.17 (Li 2013), followed by quality filtering with Samtools v1.2 (Li et al. 2009), applying thresholds for mapping quality (< 20), multiple best hits, unmapped reads, and adjusted quality (< 50). Variant detection was executed using GATK v4.0.11.0 (van der Auwera and O'Connor 2020), specifically the HaplotypeCaller module, with parameters set to a minimum base quality of 20, output mode EMIT_ALL_ACTIVE_SITES, and ERC = GVCF. The resulting GVCF files from all samples were merged using GenomicsDBImport, and subsequent genotyping, filtering, and refinement processes were applied through GenotypeGVCFs, SelectVariants, and VariantFiltration. The filtering criteria included QD < 2.0, MQ < 40.0, FS > 60.0, SOR > 3.0, MQRankSum < 12.5, and ReadPosRankSum < 8.0.

For phylogenetic reconstruction, a maximum likelihood tree was constructed from the filtered biallelic SNP dataset using IQ‐TREE v2.0 (Nguyen et al. 2015). Population genetic structure was inferred through Bayesian clustering analysis of unlinked SNPs implemented in ADMIXTURE v1.23 (Alexander et al. 2009), with the optimal number of genetic clusters (*K* = 2–8) determined by minimizing cross‐validation error. Gene flow analyses were carried out using three complementary approaches: (1) Maximum likelihood trees with 0–7 migration edges were constructed using TreeMix v1.13 (Pickrell and Pritchard 2012), with three independent runs conducted for each number of migration edges; the optimal m value was selected based on the variance‐explained curve and log‐likelihood values; (2) residual heatmaps and phylogenetic trees were visualized using the bundled scripts treemixVarianceExplained.R and draw_treemix.R, respectively; (3) the Dtrois module in Dsuite v0.5 r50 (Malinsky et al. 2021) was employed to characterize introgression patterns by analyzing SNP topologies among an outgroup (O) and three target populations (P1–P3).

## 3 Result

### 3.1 Taxonomic Treatment

**FIGURE 2:**
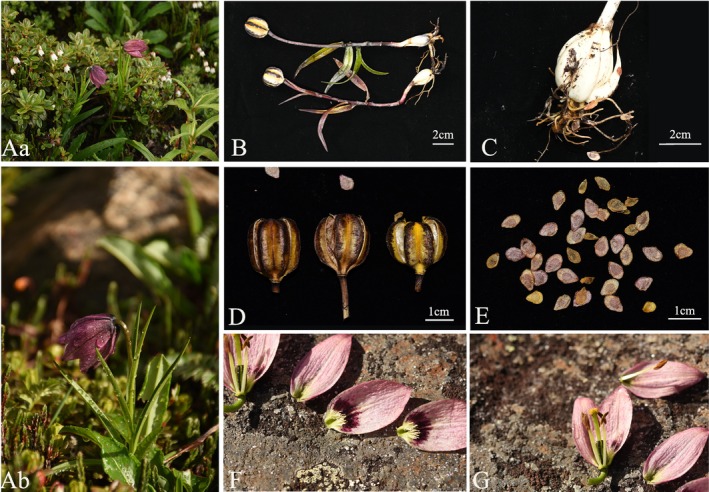
Photographs illustrating the key features of *L. pseudonanum*. (A) Habitat; (B) Plant bearing fruit; (C) Bulb; (D) Fruit; (E) Seed; (F) Anatomical view of the flower; (G) Style and stigma.

***Lilium pseudonanum*** X.J.Chen & Y.D.Gao, **sp. nov**. (Figure 2)

**若小百合 ruo xiao bai he**

#### 3.1.1 Etymology

The specific epithet “pseudonanum” is derived from the Latin word “pseudo” meaning “false” or “similar to”, and “nanum” referring to *L. nanum* , together the epithet indicating the high resemblance to the latter.

#### 3.1.2 Type

CHINA. Xizang, July 2nd 2022, Y. Gao & Y. Yuan, *GYD01404*, (holotype CDBI0286939, isotype CDBI0286940).

#### 3.1.3 Description

Bulb oblong, 2.0–3.5 cm in diam, scales white, lanceolate, 2–2.5 cm × 5–8 mm. Stem 10–15 cm. Leaves scattered, narrow lanceolate to linear, 6–11 cm × 6–12 mm, leaves size gradually decreasing from base to upper part. Flower solitary, nodding, campanulate. Tepals pale purple or whitish green; outer ones elliptic, 2.5–2.7 × 1.2–1.5 cm, nectaries glabrous and dark green in color; inner ones slightly wider than outer, and more oval in shape, usually with deep purple spots forming a purple‐red blotch adaxially, nectaries greenish with fimbriate projections (like coral) on both surfaces. Stamens converging and shorter than the style; filaments 1–12 mm, glabrous; anthers brown, ca. 6 mm. Ovary ca. 8–10 mm × 3–5 mm, tinged purple on ribs. Style 10–12 mm; stigma three‐lobed, inflated, 3–4 mm in diam. Capsule 1.9–2.5 × 2–2.5 cm. Fl. Jun‐Jul, fr. Sep.

#### 3.1.4 Habitat and Distribution

On moist bushy and grassy slopes; ca. 3900–4200 m. SE Xizang (Milin[米林]).

#### 3.1.5 Conservation Status

Investigations conducted from the summer of 2022–2024 indicated that *L. pseudonanum* was located at a single site, with approximately 100 mature individuals recorded annually. Consequently, the species' Extent of Occurrence (EOO) is assessed to be 0 km^2^ due to its single distribution, while its Area of Occupancy (AOO) is estimated at around 4 km^2^. During our field surveys, we observed that the species' habitat is frequently affected by avalanches, which further disrupt its population size and phenological patterns. Given its restricted distribution, small population size, and unstable habitat conditions, we recommend classifying *L. pseudonanum* as Critically Endangered (CR, B2i + ii, C2) according to the IUCN Red List criteria (IUCN 2024), necessitating immediate conservation actions.

### 3.2 Morphological Analysis

#### 3.2.1 Comparison With Related Species

Morphological comparisons were conducted between the new species and closely related species, particularly those that cluster closely on the nuclear gene phylogenetic tree, including *Lilium fargesii*, *L. stewartianum*, and *L. matangense*, as phenotypic differences are often more closely associated with biparental inherited nuclear genes. Although *L. pseudonanum* is closely related to the aforementioned species on the nuclear phylogenetic tree, it exhibits greater morphological divergence, particularly in traits such as plant height and flower shape and color (see Table 1). In contrast, the new species displays phenotypic similarities to *L. nanum* , warranting a more detailed comparative analysis as presented below.

**TABLE 1:** Morphological comparisons of *L. pseudonanum*, *L. fargesii*, *L. stewartianum*, and *L. matangense*.

Character	*L. pseudonanum*	*L. matangense*	*L. stewartianum*	*L. fargesii*
Bulb oblong	2.0–3.5 cm	1.0–1.5 cm	ca. 2 cm	ca. 1.5 cm
Stem	10–15 cm	23–35 cm	20–50 cm	20–70 cm
Leaves				
Shape	Scattered, narrow lanceolate to linear	Scattered, linear	Scattered, linear	Scattered, mostly in middle and distal parts of the stem, linear
Size	6–11 × 0.6–1.2 cm	6–11 × 0.1–0.4 cm	2.5–7 × 0.3–0.4 cm	10–14 × 0.2–0.5 cm
Flower				
Shape	Campanulate	Tepal‐reflexed	Tepal‐reflexed	Tepal‐reflexed
Perianth plate color	Purple or whitish green; outer ones elliptic	Tepals white, tinged pale brown, with purple‐brown spots	Tepals greenish yellow, with deep red spots	Tepals greenish white, with dense, purple, or purple‐brown spots
Perianth plate size	2.5–2.7 × 1.2–1.5 cm	2.5–3.5 × 0.5–0.7 cm	4.5–5 × 0.7–0.9 cm	3–3.5 × 0.7–1 cm
Ovary	0.8–1 × 0.3–0.5 cm	1–1.5 × ca. 0.2 cm	2–2.2 × ca. 0.3 cm	1–1.5 × ca. 0.2 cm
Capsule	1.9–2.5 × 2–2.5 cm	1–1.3 cm	2–2.5 × 1.5–2 cm	2 × 1.5 cm

#### 3.2.2 Morphological Trait Data Analysis

A total of 13 quantitative traits were selected for analysis based on morphological characteristics. The one‐way ANOVA conducted on *L. pseudonanum* and *L. nanum* revealed no significant differentiation between the two taxa. However, the data indicate that the basal leaves and fruits of *L. pseudonanum* are larger in size compared to those of *L. nanum* (see Table 2).

**TABLE 2:** Morphological comparison based on measurements of multiple individuals between the *L. pseudounanum* and *L. nanum* .

	*Lilium pseudounanum*	*Lilium nanum*
Plant height	19.44 ± 3.60	18.87 ± 1.30
Basal leaf length	5.43 ± 0.98	3.94 ± 0.28
Basal leaf width	0.66 ± 0.07	0.33 ± 0.02
Middle leaf length	6.52 ± 1.23	6.16 ± 0.31
Middle leaf width	0.63 ± 0.09	0.27 ± 0.02
Apical leaf length	5.84 ± 1.03	5.88 ± 0.33
Apical leaf width	0.39 ± 0.10	0.19 ± 0.02
Fruit length	2.47 ± 0.21	2.13 ± 0.13
Fruit width	1.95 ± 0.11	1.65 ± 0.11
Fruit area	4.35 ± 0.76	2.96 ± 0.33
Shape	Purplish red	Purplish red

The cumulative contribution rate of the first four principal components derived from the 13 morphological features accounted for 80.80% of the total variance, with the first principal component (PC1) and second principal component (PC2) explaining 30.21% and 21.50% of the variation, respectively. PC1 was primarily associated with plant height, basal leaf length, basal leaf width, middle leaf length, middle leaf width, apical leaf length, apical leaf width, fruit length, fruit width, and fruit area. Conversely, PC2 was mainly associated with plant height, basal leaf length, the length‐to‐width ratio of basal leaves, middle leaf length, middle leaf width, apical leaf length, and apical leaf width (Table 3).

**TABLE 3:** PCA analysis factor loadings based on 13 morphological characteristics.

	PC1	PC2	PC3	PC4
PH	**0.594**	**−0.578**	0.255	**−0.139**
BL	**0.449**	**−0.450**	0.418	0.370
BW	**0.649**	0.002	**−0.270**	**−0.401**
BR	−0.145	**−0.475**	0.515	0.637
ML	**0.631**	**−0.289**	0.518	**−0.124**
MW	**0.630**	**−0.370**	**−0.521**	**−0.032**
MR	−0.116	0.396	0.771	**−0.066**
TL	**0.626**	**−0.334**	0.419	**−0.245**
TW	**0.754**	**−0.164**	**−0.449**	0.163
TR	−0.125	0.152	0.726	**−0.455**
FL	**0.613**	0.689	0.149	0.182
FW	**0.529**	0.756	0.040	0.180
FA	**0.699**	0.669	0.082	0.180

*Note:* Values in the table with factor loadings > 0.3 and < −0.3 are bolded.

Abbreviations: BL, basal leaf length; BR, basal leaf length‐to‐width ratio; BW, basal leaf width; FA, fruit area; FL, fruit length; FW, fruit width; ML, middle leaf length; MR, middle leaf length‐to‐width ratio; MW, middle leaf width; PH, plant height; TL, top leaf length; TR, top leaf length‐to‐width ratio; TW, top leaf width.

**FIGURE 3:**
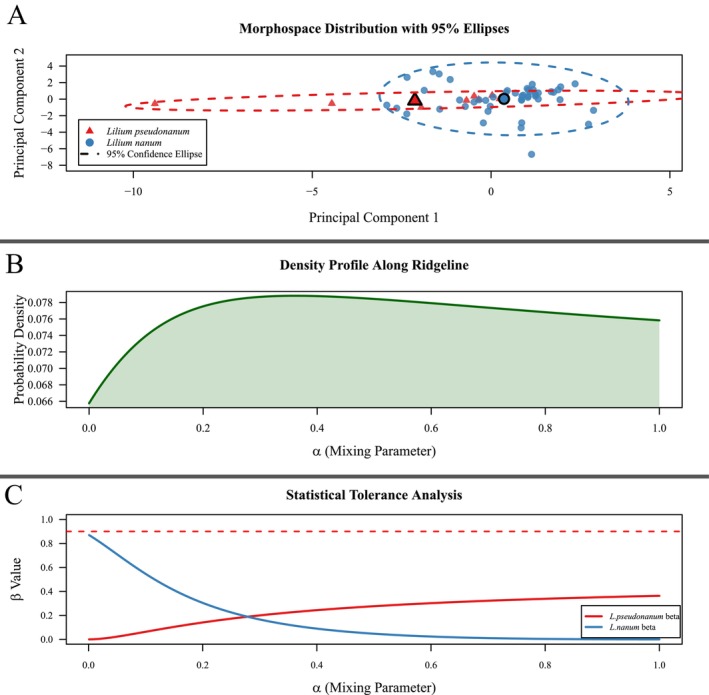
Inferences of morphological gaps between *L. nanum* and *L. pseudonanum*. (A) PCA illustrating the pattern of morphological variation between the two putative species. (B) Estimated probability density function evaluated at various points along the ridgeline manifold (α), representing the mixture of two normal distributions of morphological variation suggested by the hypothesized species boundary. (C) Estimated proportion (β) (on the ordinate) encompassed by the elliptical tolerance regions (γ = 0.95) that converge at a single point (α) on the ridgeline manifold.

The morphological divergence between *L. nanum* and *L. pseudonanum* was quantitatively assessed through multivariate analyses. PCA revealed a continuous distribution of both groups within the PC1–PC2 morphospace, with no statistically significant clustering observed (Figure 3A). Saddle‐line manifold analysis demonstrated a unimodal distribution of probability density functions along the interspecific mean vector (Figure 3B), failing to meet the criteria for morphological discontinuity as defined by Ray and Lindsay (2005). These results suggest that the current morphometric data do not support the recognition of *L. nanum* and *L. pseudonanum* as distinct species, further emphasizing the cryptic nature of the latter in comparison with *L. nanum* .

### 3.3 Phylogenetic Analysis

In this study, a phylogenetic analysis was conducted to investigate the evolutionary relationships among different species. Two datasets, comprising ITS sequences and chloroplast genomes, included a total of 43 sequences, which featured two individuals of *Lilium pseudonanum* and four individuals of *L. nanum* . Sequences for the other species were downloaded from the National Center for Biotechnology Information (NCBI) (https://www.ncbi.nlm.nih.gov/). The ITS sequence of the new species has a length of 626 bp and a GC content of 61.98%, while the chloroplast genome sequence measures 152,070 bp in length with a GC content of 36.99%. The lengths of the ITS sequences varied from 541 bp to 638 bp, and the overall alignment length was 647 bp, comprising 244 variable sites and 392 conserved sites. For the chloroplast genomes, lengths ranged from 151,058 bp to 153,235 bp. Following alignment correction, the total sequence length was standardized to 159,091 bp, which included 7966 variable sites and 148,784 conserved sites.

**FIGURE 4:**
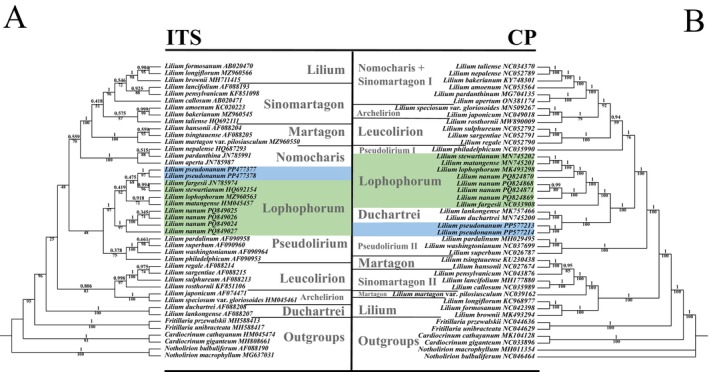
Molecular phylogenetic tree conducted. (A) Nuclear ITS; (B) chloroplast whole genome. Both trees were based on the maximum likelihood (ML) method and Bayesian inference and included 43 species as representatives for the major clades of the genus *Lilium*. The branch support values in the ML tree are displayed below the corresponding nodes, while the BI values are shown above the nodes. Green represents clade Lophophorum, and blue represents the new species.

The phylogenetic analysis based on ITS sequences indicated that the putative new species, *L. pseudonanum*, is closely related to *L. fargesii* Franch. and *L. stewartianum* Balf. f. et W.W. Sm. However, due to insufficient informative sites in the ITS data, support for this relationship is low, with a maximum likelihood (ML) value of only 68% and a bootstrap likelihood (BL) of 0.475. Together, these species belong to a clade that includes members of, although the putative new species is not allied with *L. nanum* . This clade further associates with another clade comprising *L. duchartrei* and *L. lankongense*, which is robustly supported by a 100% confidence level (Figure 4).

Conversely, the chloroplast phylogenetic tree exhibited a different pattern. Unlike the ITS analysis, where *L. pseudonanum* formed a clade with members of Lophophorum, in the chloroplast dataset, *L. pseudonanum* was found to be closely related to *L. lankongense* and *L. duchartrei*, forming a distinct clade with strong support of 100%. Following annotation, it was determined that *L. pseudonanum* contains 134 genes and 84 coding sequences (CDS). After correcting alignments for all coding sequences, the total length was calculated to be 82,357 base pairs, consisting of 2992 variable sites and 77,519 conserved sites. The phylogenetic tree constructed from the plastome CDS aligns well with these findings obtained from the full‐length analysis (Figure S3). The Duchartrei clade is sister to the Lophophorum clade, with a high confidence level of 100%. Although discrepancies exist between the CDS and complete chloroplast datasets, both analyses indicate that *L. pseudonanum* is not directly related to *L. nanum* .

### 3.4 Chloroplast Genome Synteny Analysis

**FIGURE 5:**
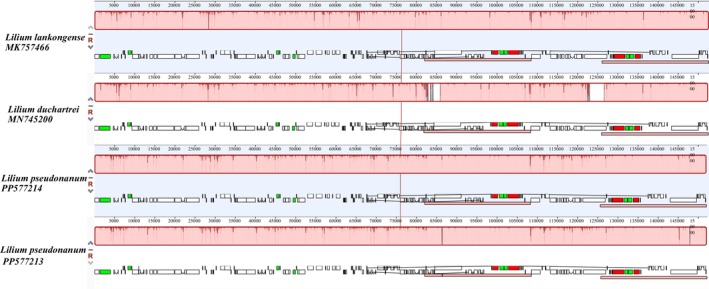
Synteny analysis of the chloroplast genomes between *L. pseudonanum*, *L. lankongense* and *L. duchartrei*.

Synteny analysis of chloroplast genome was conducted among *Lilium pseudonanum*, *L. lankongense*, and *L. duchartrei*. The results from the Mauve multigenome alignment (Figure 5) indicate that the chloroplast genomes of these three species share two locally collinear blocks (LCBs), with no evidence of large‐scale rearrangements or inversions. These genomes demonstrate high levels of similarity and collinearity, further supporting the close relationship among the aforementioned species at the chloroplast structural level, in addition to the phylogenetic inferences.

### 3.5 Division of Potential Habitats of *Lilium nanum*

**FIGURE 6:**
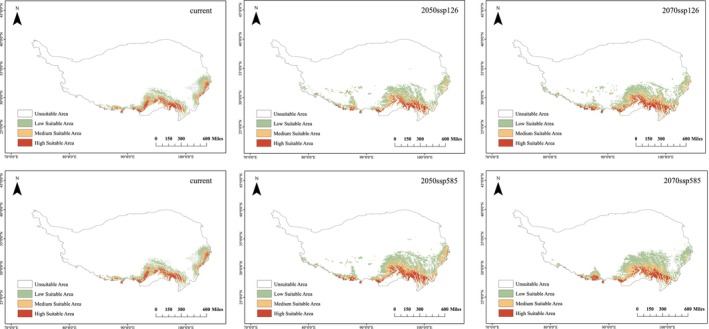
Assessment of suitable habitats for *L.* *nanum* under climate scenarios SSP126 and SSP585. Habitat suitability is classified as follows: scores of [0, 0.24] indicate nonsuitable habitat; [0.24, 0.48] indicate low suitable habitat; [0.48, 0.84] indicate moderate suitable habitat; and [0.84, 0.99] indicate high suitable habitat.

The areas of *Lilium nanum* across various levels of habitat suitability under different climate scenarios were quantified and represented on the map (Figure 6). Suitable habitats for *L. nanum* are continuously distributed across the Xizang, Sichuan, and Yunnan provinces, with a predominant concentration in the alpine regions of the Himalayas and the Hengduan Mountains. Under future climate change scenarios, by the 2050s, the suitable habitat for *L. nanum* is projected to shift significantly eastward relative to its current distribution, resulting in an overall expansion of the suitable habitat range. However, the area characterized by high suitability is expected to decline, accompanied by a movement to higher altitudes. By the 2070s, there is anticipated to be minimal significant change in suitable habitats compared to present conditions, although a trend of expansion toward the southwest in high suitability areas is evident.

### 3.6 Inference of Potential Paleohybridization and Introgression Events

The phylogenetic incongruence observed between the nuclear ITS and chloroplast gene trees (Figure 3), particularly the discordant placement of *Lilium pseudonanum*, prompted an integrated analysis of eight representative species (in total 12 accessions). This analysis included the focal taxa (*L. pseudonanum* and *L. nanum* ), representatives of the recurved tepal clade (*L. lophophorum*), the campanulate flower clade (*L. fargesii*, *L. matangense*, *L. stewartianum*), and representatives from the Duchartrei lineage (two accessions), along with two outgroup species ( *L. regale* and *L. henryi* Baker, one accession each). The application of GRAMPA‐based multilabel tree reconciliation (Figure S4) resolved *L. nanum* and *L. pseudonanum* into two distinct phylogenetic branches. One branch, which includes *L. pseudonanum*, clustered with *L. lophophorum* based on nuclear genomic data, yet the chloroplast sequences were nested within the Duchartrei clade (Figure 4). Conversely, the second branch, comprising *L. nanum* , exhibited congruent nuclear and chloroplast signals that support independent divergence. This nuclear‐plastid discordance—indicating nuclear evidence for hybridization coupled with chloroplast replacement for maternal heritage—suggests historical hybridization accompanied by plastid introgression.

### 3.7 Results of Population Genomic Analyses

**FIGURE 7:**
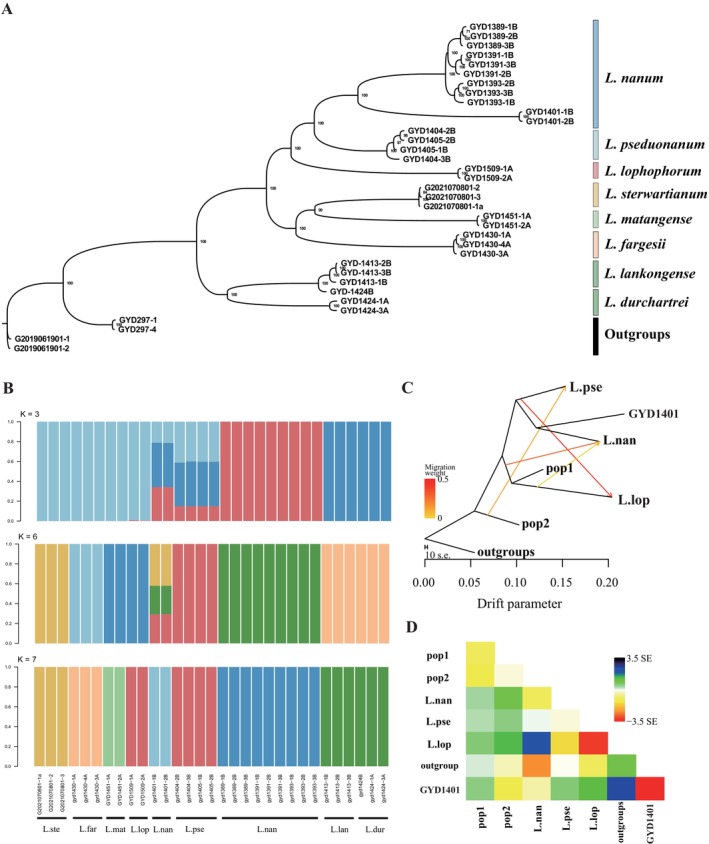
Population genetic structure and phylogenetic relationships. (A) Comparison of topologies inferred from nuclear SNP; (B) Genetic structure inferred by ADMIXTURE (*K* = 3, 6, 7); (C) TreeMix gene flow phylogeny (M = 4). (D) TreeMix residual plot. pop1 includes campanulate‐flowered species *L. fargesii*, *L. matangense*, and *L. stewartianum*, while pop2 includes representatives of the Duchartrei lineage.

Building on the nuclear‐plastid discordance unveiled by earlier ITS/chloroplast phylogenies and GRAMPA analyses, this study expanded the sampling to eight taxa (35 accessions) and two outgroups, consistent with GRAMPA analysis. The dataset comprised focal taxa, with *Lilium pseudonanum* (*n* = 4) and *L. nanum* (*n* = 11), allied recurved tepal species *L. lophophorum* (*n* = 2), campanulate flower species (*L. fargesii*, *n* = 3; *L. matangense*, *n* = 2; *L. stewartianum*, *n* = 3), Duchartrei lineage representatives (*n* = 6), and outgroups *L. regale* and *L. henryi* (*n* = 2 for each). A maximum likelihood (ML) tree constructed from biallelic SNPs revealed strongly supported monophyletic clades (bootstrap > 90%), with *L. pseudonanum* forming an independent branch, reinforcing its status as a cryptic species (Figure 7A). However, the *L. nanum* clade exhibited significant genetic heterogeneity, demonstrated by complex internal branching patterns in sample GYD1401, suggestive of hybrid introgression.

ADMIXTURE analysis (Figure 7B) indicated tripartite admixture in *L. pseudonanum* at *K* = 3, encompassing the Lophophorum clade, Duchartrei clade, and *L. nanum* . The optimal *K* = 6 model revealed clear species differentiation, although the individual GYD1401 retained admixed ancestry involving *L. pseudonanum* and *L. stewartianum*. Complete lineage sorting at *K* = 7 further validated the taxonomic robustness. TreeMix analysis (Figure 7C) with four migration edges (M = 4, FDR < 0.05) uncovered strong gene flow signals from *L. lophophorum* to *L. pseudonanum* and from the Duchartrei clade (pop2) to *L. pseudonanum*. ABBA‐BABA tests corroborated these findings; the critical contrast between *L. pseudonanum* and the Duchartrei clade (pop1/*L. lop*/*L. pse*) yielded a highly significant positive D‐statistic (D = 0.1166, *Z* = 15.76, *p* < 0.01), with an ABBA/BABA site ratio (f4‐ratio = 0.123) strongly supporting chloroplast capture from Duchartrei in conjunction with nuclear introgression from the Lophophorum clade. Additionally, gene flow between *L. pseudonanum* and *L. lophophorum* was detected at lower but significant levels (D = 0.0236, *Z* = 2.95, *p* < 0.01), aligning with TreeMix inferences of bidirectional contributions (Table 4). Collectively, these multilayered genomic signatures—chloroplast discordance, directional gene flow, and asymmetrical ABBA‐BABA signals—support the hypothesis that *L. pseudonanum* originated from ancient hybridization.

**TABLE 4:** ABBA‐BABA test results.

P1	P2	P3	D‐statistic	*Z*‐score	*p* value	F4 ratio	BBAA	ABBA	BABA
GYD1401	L.nan	L.lop	0.00308579	0.369102	0.712052	0.00174733	5877.3	3714.65	3691.8
L.pse	GYD1401	L.lop	0.017972	2.27389	0.0229726	0.0119824	4750	4439.19	4282.44
GYD1401	L.lop	pop1	0.0758663	9.7145	2.3e−16	0.118397	4927.49	4779.22	4105.19
GYD1401	L.lop	pop2	0.0106965	1.15728	0.247158	0.0113625	7458.7	3325.12	3254.74
L.nan	GYD1401	L.pse	0.0550863	5.93815	2.88258e−09	0.0566096	5702.08	3850.33	3448.28
GYD1401	L.nan	pop1	0.0311355	3.85562	0.000115436	0.0403276	6677.43	3846.18	3613.9
GYD1401	L.nan	pop2	0.0275163	3.69973	0.000215829	0.0252869	9268.27	2963.98	2805.23
L.pse	GYD1401	pop1	0.0429272	4.99705	5.82127e−07	0.0596947	5511.98	4380.08	4019.5
GYD1401	L.pse	pop2	0.0369419	4.77861	1.76511e−06	0.0377103	7547.91	3273.65	3040.39
pop1	GYD1401	pop2	0.00636851	0.687113	0.492012	0.00694487	6911.01	3472.72	3428.77
L.lop	L.nan	L.pse	0.00723081	1.07525	0.282265	0.00901077	4621.66	4508.23	4443.5
L.nan	L.lop	pop1	0.0525228	7.83479	4.69622e−15	0.085709	4941.36	4769.17	4293.19
L.lop	L.nan	pop2	0.00762567	1.35378	0.175806	0.00846267	7584.11	3466.44	3413.97
pop1	L.lop	L.pse	0.116581	15.7621	2.3e−16	0.122919	4887.43	4839.35	3828.81
L.lop	L.pse	pop2	0.0235523	2.95048	0.00317276	0.0246064	7083.56	3271.2	3120.65
pop1	L.lop	pop2	0.0193917	3.09725	0.00195323	0.0191996	7280.45	3204.53	3082.61
L.pse	L.nan	pop1	0.0648175	13.7787	2.3e−16	0.095082	5302.16	4781.17	4199.09
L.nan	L.pse	pop2	0.0117104	1.98558	0.0470805	0.0128124	7284.94	3381.18	3302.91
pop1	L.nan	pop2	0.0263325	4.34236	1.4096e−05	0.0299615	7299.37	3752.84	3560.26
pop1	L.pse	pop2	0.0390485	6.64054	3.12545e−11	0.0416024	6354.6	3501.86	3238.65

*Note:* pop1 includes campanulate‐flowered species *L. fargesii*, *L. matangense*, and *L. stewartianum*, while pop2 includes representatives of the Duchartrei lineage.

## 4 Discussion

Traditionally, the appearance of the perigone has been regarded as the most critical characteristic for identifying lilies. Based on this premise, the genus *Lilium* is categorized into four major floral morphological types: tepal‐reflexed, campanulate, trumpet, and flat open (Liang and Tamura 2000; Givnish et al. 2020). Historically, these morphological characteristics have served as key factors for subgeneric classification (Wilson 1925; Comber 1949; Baranova 1988). However, recent phylogenetic studies suggest that such classifications may not always provide accurate reflections of evolutionary relationships and can sometimes be misleading due to parallel evolution, which results in superficial affinities (Gao, Harris, et al. 2013; Gao et al. 2015; Du et al. 2014; Zhou et al. 2020; Yuan and Gao 2024). This indicates that reliance solely on morphological data may not adequately resolve relationships within *Lilium* and, therefore, such data should be interpreted with caution. The variety in floral morphology is presumably linked to significant ecological differences across geographic distributions. Consequently, using morphological traits exclusively to clarify interspecific relationships in *Lilium* can be unreliable, particularly in light of the prevalent parallel evolution within the genus (Gao et al. 2015; Yuan and Gao 2024; Feng et al. 2025; Duan et al. 2025); thus, distinguishing subgeneric groups using shared traits proves problematic. The newly identified species in this study exemplifies these challenges.

As a case of a morphologically complex group, the Lophophorum clade encompasses species that inhabit the high mountainous regions of southwestern China. This clade is characterized by dwarf stems, linear or lanceolate leaves, and typically bell‐shaped flowers, as delineated within the monophyletic framework of Lophophorum sensu Gao, Harris, et al. (2013). Notably, alongside typical campanulate forms, certain species like *L. fargesii* exhibit recurved tepals. Members of this monophyletic clade have a wide distribution, extending from the broad‐leaved forests of the Daba Mountain Range in central China to high‐altitude shrublands in the southwestern Hengduan Mountains and further to the alpine shrub meadows of the Himalayas (Wu et al. 2014; Wang et al. 2025). The range of the Lophophorum clade encompasses a significant geographic span from central China (e.g., *L. fargesii*) in the east to the western Himalayas (e.g., *L. nanum* ) in the west (Figure 1). In the western part of this range, *L. nanum* predominates in alpine meadows and has a considerable distribution extent. Its dwarf habit, nodding bell‐shaped flowers, and purplish color are likely adaptations to the harsh conditions of alpine environments, such as extreme wind and precipitation, both of which may adversely affect flower development during blooming.

The extensive range of *L. nanum* is accompanied by a wide array of variations in growth habit and organ size. For example, the variability in flower size and color raises questions regarding taxonomic classifications. A detailed examination of this topic can be found in Gao (2023). In summary, *L. nanum* can exhibit purplish and yellow flowers, with considerable variation in perigone size. Additionally, the yellow‐flowered *L. nanum* var. *flavidum* (Rendle) Sealy, previously accepted by the Flora of China (Liang and Tamura 2000), has been identified as a misclassification; thus, the name *L. euxanthum* (W. W. Smith & W. E. Evans) Sealy needs to be reinstated (Gao Yundong, unpublished data). The confusion stemmed from the misleading morphological similarities, which are adaptations to similar alpine environments, a phenomenon frequently observed within the genus *Lilium* (Gao et al. 2015; Yuan and Gao 2024; Feng et al. 2025). The complex status and history of *L. nanum* suggest that a more comprehensive sampling strategy, employing additional lines of evidence beyond morphology, is necessary to uncover hidden aspects important for understanding the diversification of this species and related taxa.

This study contributes to the ongoing exploration of the aforementioned challenges. Advances in molecular technology have revolutionized systematic classification by facilitating the integration of phenotypic and genotypic data, thereby enhancing taxonomic resolution (Huang et al. 2018; Ran et al. 2018; Yuan et al. 2025). In the current investigation, molecular phylogenetic analysis utilizing nuclear ribosomal DNA (ITS) places *L. pseudonanum* within the Lophophorum clade, demonstrating a close relationship with *L. fargesii* and *L. stewartianum*, both of which possess recurved tepals. Conversely, chloroplast data indicate that this new species aligns more closely with a different clade that includes *L. duchartrei* and *L. lankongense* (Duchartrei‐clade *sensu* Gao, Harris, et al. 2013). While there is not complete congruence between the chloroplast and nuclear phylogenetic trees regarding the positioning of *L. pseudonanum*, it is unequivocally established that this species is distinct from *L. nanum* —regardless of their morphologically similar traits—and other members within the Lophophorum clade, confirming its independent status in a genetic sense.

Distinguishing the morphological similarities between the new species and *L. nanum*—such as elongated bulbs, linear leaves, dwarf growth habits, and solitary nodding bell‐shaped flowers exhibiting greenish‐yellow or purplish colors—poses considerable challenges. Moreover, *L. pseudonanum* has a restricted distribution that is entirely encompassed within the eastern portion of the *L. nanum* range, complicating recognition of the former. While statistical morphological methods were employed to identify distinguishing features between the new species and its closest relative, PCA revealed no significant morphological differences between the two (Figure 3A). Field surveys, in conjunction with ANOVA (Table 1), indicated that the key distinguishing features of *L. pseudonanum* include its larger basal leaves. However, this trait alone is insufficient for clear delimitation as a quantitative characteristic. PCA analysis identified basal leaf length and width as crucial factors for differentiating the two taxa, given that these traits significantly influenced both PC1 and PC2 (Table 3). Nonetheless, due to the extensive distribution range of *L. nanum* and its associated morphological variability, isolating this feature and recognizing further distinctions becomes increasingly challenging. Furthermore, morphological traits often exhibit plasticity influenced by environmental conditions, rendering them unreliable indicators (Givnish et al. 2020). The lack of morphological differentiation between *L. nanum* and the new species, along with the molecular inferences mentioned above, suggests that the new species is cryptic in nature.

In verifying the status of the cryptic species, the integrated species concept—advocating for the synthesis of multiple lines of evidence to define species—could prove effective, especially as an increasing number of researchers recognize that species boundaries may be maintained by various factors, including ecological ones (Liu 2016; Donoghue 2022; Yuan et al. 2025). Recent findings in the genus *Lilium* suggest that niche diversification plays a crucial role in upholding species boundaries among closely related taxa (Feng et al. 2025). In the current study, *L. nanum* is found to inhabit a range spanning from the western Himalayas to the Hengduan Mountains, predominantly in alpine meadows and shrublands at elevations around 4000 m (Su et al. 2021; Gao 2023; Wang et al. 2025). Its optimal growth conditions are compromised under excessively low precipitation during the coldest quarter, high elevations, or when annual mean temperatures and precipitation exceed optimal levels. Specifically, precipitation during the coldest quarter significantly affects its growth (Figure S5C), indicating that *L. nanum* is particularly sensitive to changes in moisture availability, which in turn impacts its transpiration and, consequently, leaf growth and overall area.

In contrast, *L. pseudonanum* thrives on mountain peaks in southeastern Xizang, benefiting from increased precipitation due to its position facing the Indian Ocean, particularly during the summer and winter months. Data from Chu (2022) reveal that 20 snow‐related disasters were recorded in Metok (Medog) County from 2015 to 2019, constituting 18.2% of the total avalanches reported in Xizang. The habitat of the new species experiences perennial snow cover and heightened moisture levels during both winter and summer, resulting in greater humidity and precipitation compared to that of *L. nanum* . For instance, meteorological observations indicated that snowfall intensity reached approximately 86.9 mm on December 26, 2022, with total winter snowfall exceeding 100 mm, compared with the key environmental factors for *L. nanum* which include a precipitation range of 4.22–93.86 mm during the Coldest Quarter (Figure S5C). Despite both species occurring at similar altitudes, they occupy distinct ecological niches, even when geographically proximate. *L. pseudonanum* is found in environments characterized by higher humidity and prolonged snow cover, which may contribute to its more substantial and compact leaf structures. PCA reveals a trend toward differentiation in leaf length and width between the two species, suggesting that seasonal humidity contrasts are critical environmental factors driving their divergence. Consequently, niche diversification likely plays a pivotal role in the speciation of this cryptic species.

The origin of *L. pseudonanum* warrants further exploration beyond niche differentiation from *L. nanum*. Phylogenetic analyses revealing cryptic species provide insights into their potential origins. The discrepancies noted in two molecular datasets suggest a possible hybrid origin. Hybridization and gene introgression are major contributors to nuclear‐cytoplasmic conflicts in lilies (Gao, Harris, et al. 2013; Gao et al. 2015, 2020; Zhou et al. 2020; Yuan and Gao 2024). The nuclear ITS phylogeny places *L. pseudonanum* definitively within the Lophophorum clade, corroborated by previous studies affirming the reliability of ITS phylogeny for resolving sect‐level relationships within the genus *Lilium* (Gao, Zhou, and He 2013; Gao et al. 2015). While chloroplast genomes provide additional data, their maternal inheritance and historical hybridization events can obscure phylogenetic clarity due to geographic preferences (Gao, Zhou, and He 2013). Typically, closely related species are geographically separated, while sympatric species often exhibit distant phylogenetic relationships (Weir and Price 2011). The molecular discrepancies imply that *L. pseudonanum* may have arisen from hybridization or historical gene flow between the Lophophorum and Duchartrei clades (Figure 4), which exhibit overlapping distributions in the western Hengduan Mountains, a biodiversity hotspot (Figure 1).

The chloroplast genome of *L. pseudonanum* displays substantial similarity and collinearity to that of the Duchartrei clade, with no evidence of large‐scale rearrangements or inversions (Figure 5). This structural consistency strongly supports a homologous origin, effectively ruling out alternative mechanisms such as incomplete lineage sorting (ILS) that would typically manifest as disordered patterns. Though the ITS phylogeny does not resolve the relationship between the Lophophorum and Duchartrei clades, recent transcriptome‐based phylogenetic analyses (Liang et al. 2025) indicate a sister relationship between these clades. This genetic proximity suggests potential compatibility for interspecific hybridization, thus supporting the hypothesis that the Duchartrei clade may have acted as the maternal progenitor of *L. pseudonanum*.

Geographically, the Duchartrei clade overlaps with the range of several species from the Lophophorum clade across the southeastern Tibetan Plateau, enhancing opportunities for hybridization (Figure 1). This ecological context provides a viable mechanism for the transfer of maternal genotypes between species. The multilayered evidence for the hybrid origin of *L. pseudonanum* includes nucleocytoplasmic discordance (nuclear ITS vs. chloroplast), phylogenetic incongruence, and biogeographic range overlap. GRAMPA‐based multilabel tree reconciliation (Figure S4) substantiates this hypothesis, positioning *L. pseudonanum* within the Lophophorum clade based on nuclear genomic data, while its chloroplast genome nests within the Duchartrei clade.

The observed nuclear‐plastid discordance—showing nuclear evidence for hybridization alongside chloroplast evidence for maternal capture—indicates that *L. pseudonanum* likely originated from historical hybridization accompanied by plastid introgression. The chloroplast collinearity with the Duchartrei clade (Figure 5) and nuclear divergence from the Lophophorum clade (Figure 4) suggest a mechanism of unidirectional maternal plastid capture paired with paternal nuclear genome recombination, corresponding with recognized hybridization patterns in plants, particularly the nuclear‐plastid discordance seen in allopolyploidy or ancient introgression events (Soltis and Soltis 2009).

Population genomic analyses provide critical support for the hybrid origin hypothesis. ADMIXTURE analysis shows tripartite admixture in *L. pseudonanum* at *K* = 3, reflecting contributions from the Lophophorum clade, Duchartrei clade, and *L. nanum* . The optimal *K* = 6 model indicates clear species differentiation, although sample GYD1401 retains admixture evidence involving *L. pseudonanum* and *L. stewartianum* (from the tepal‐recurved relative), suggesting ongoing introgression within the *L. nanum* lineage (Figure 7B). Moreover, TreeMix analysis (with four migration edges; M = 4) reveals strong gene flow from the recurved tepal clade (*L. lophophorum*) and the Duchartrei clade (pop2) to *L. pseudonanum* (Figure 7C). These findings are reinforced by ABBA‐BABA tests, confirming the unidirectional transfer of chloroplasts from the Duchartrei clade and nuclear introgression from the Lophophorum clade. Collectively, these genomic signatures indicate that *L. pseudonanum* likely originated from ancient hybridization during the Late Miocene in a secondary contact zone within the southeastern Qinghai‐Tibet Plateau. Hybridization can lead to various outcomes including hybrid speciation, introgression, reinforcement, extinction of rare species, and merging of parental species (Abbott et al. 2013; Todesco et al. 2016). The newly described *L. pseudonanum* will likely encounter these challenges if a stable population is not established and maintained (Irwin and Schluter 2021). The species' habitat preferences discussed above may represent a well‐defined niche innovation, enhancing its persistence through spatial separation from parental species, particularly the widespread *L. nanum* . Thus, adaptation to a novel and distinct environment is crucial for successful speciation.

The recognition of this new species not only contributes to our understanding of speciation within a biodiversity hotspot but also highlights a rare genetic resource among wild lilies. In the context of global warming, predictions from MaxEnt models indicate that *L. nanum* is likely to migrate to higher elevations in search of cooler habitats. This predicted migration poses potential habitat disturbances for *L. pseudonanum*. ADMIXTURE analysis further elucidates recent hybridization dynamics within the *L. nanum* clade. Specifically, sample GYD1401 exhibited significant admixed ancestry at *K* = 6, suggesting recent introgression events. This finding indicates that the cryptic species *L. pseudonanum* may face similar risks of genetic assimilation. The predicted upslope migration of *L. nanum* , as shown in MaxEnt projections (Figure 6), may exacerbate this risk, as niche overlap could facilitate secondary contact and asymmetric introgression.

According to the competitive exclusion principle, species occupying similar ecological niches may face difficulties coexisting within the same habitat, resulting in increased competition for shared resources (Gause 1934). Should these two species overlap geographically, the likelihood of interspecific hybridization may increase. Such genetic mixing has been documented in several plant species that experienced ecological shifts during the last glacial maximum (LGM), approximately 26,500 to 19,000 years ago (Clark et al. 2009). Moreover, hybridization between widely distributed and narrowly distributed species can lead to genetic assimilation and population swamping, thereby threatening the viability of narrowly distributed species (Todesco et al. 2016; Zhu et al. 2023).

*Lilium pseudonanum* also faces numerous threats to its survival, including a narrow distribution range, habitat fragmentation, and climate‐driven vertical range shifts, positioning it as a critically endangered candidate species within the alpine ecosystems of the Hengduan Mountains. Although current research has yet to clarify its reproductive ecology, traits shared among high‐altitude *Lilium* taxa suggest that it may encounter challenges similar to those faced by phylogenetically related groups. For instance, studies on *Fritillaria* species (Yıldız et al. 2022, 2024; Aslay et al. 2023) reveal that alpine plants often depend on specialized pollinators, such as bumblebees and flies. Limitations in pollinator availability and declines in pollen viability are significant factors contributing to reproductive failure.

Furthermore, the critically endangered status of the recently discovered nova *Lilium huanglongense* T. Wang & Y.D. Gao, with fewer than 30 individuals remaining in the wild, underscores the increased extinction risk faced by small, isolated alpine lily populations under extreme climatic disturbances in the genus *Lilium* (Wang et al. 2025). As a cryptic species, *L. pseudonanum* is particularly vulnerable to conservation neglect; however, its ecological importance as a keystone indicator species—vital for maintaining pollinator networks and soil microecological stability—cannot be overstated. Given the dual pressures of climate change and anthropogenic habitat degradation, *L. pseudonanum* faces an imminent risk of extinction, necessitating the urgent implementation of integrated conservation strategies.

## Author Contributions

**Yundong Gao:** writing – original draft (equal), writing – review and editing (equal), supervision, project administration, methodology, investigation, funding acquisition, conceptualization. **Xiaojuan Chen:** writing – original draft (equal), writing – review and editing (equal). **Yumei Yuan:** data curation (equal).

## Ethics Statement

The authors have nothing to report.

## Conflicts of Interest

The authors declare no conflicts of interest.

## Supporting information


Appendix S1

## Data Availability

The sequences of the newly generated chloroplast genomes were deposited in the National Center for Biotechnology Information (NCBI) GenBank database under the accession numbers PP577214 and PP577213, and the additional data can be found in the main text or made available on request.
