# Supplementary material for: Lilium pseudonanum (Liliaceae), a Rare and Cryptic Species From Southeast Xizang, China
Source: Ecol Evol. 2025 Jul 10;15(7):e71738. doi: 10.1002/ece3.71738 (PMC12245480; doi:10.1002/ece3.71738)
Supplement: Supplementary file 1 — Appendix S1 [file ECE3-15-e71738-s001.zip › FigS1.pdf]

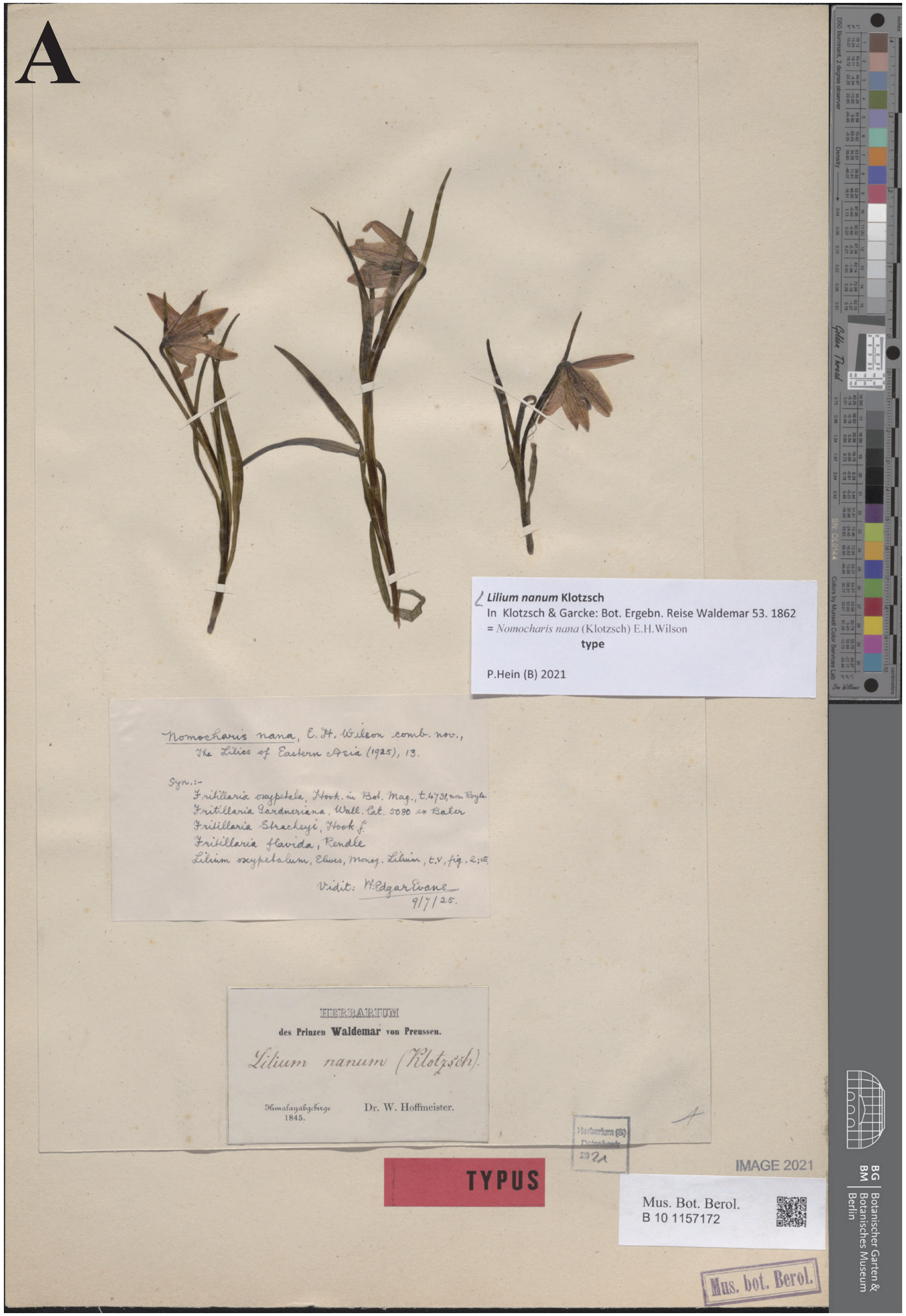
A
Lilium nanum Klotzsch
In Klotzsch & Garcke: Bot. Ergebn. Reise Waldemar 53. 1862
= Nomocharis nana (Klotzsch) E.H.Wilson
type
P.Hein (B) 2021
Nomocharis nana, E. H. Wilson comb. nov.,
The Lilies of Eastern Asia (1925), 13.
Syn.:-
Fritillaria oxypetala, Hook. in Bot. Mag., t.4731, non Royle
Fritillaria Gardneriana, Wall. Cat. 5080 ex Baker
Fritillaria Stracheyi, Hook f.
Fritillaria flavida, Rendle
Lilium oxypetalum, Elwes, Monog. Lilium, t.V, fig. 2; etc.
Vidit: W. Edgar Evans
9/7/25.
HERBARIUM
des Prinzen Waldemar von Preussen.
Lilium nanum (Klotzsch).
Himalayagebirge 1845.
Dr. W. Hoffmeister.
TYPUS
IMAGE 2021
Mus. Bot. Berol.
B 10 1157172
Mus. bot. Berol.
BG BM | Botanischer Garten & Botanisches Museum Berlin
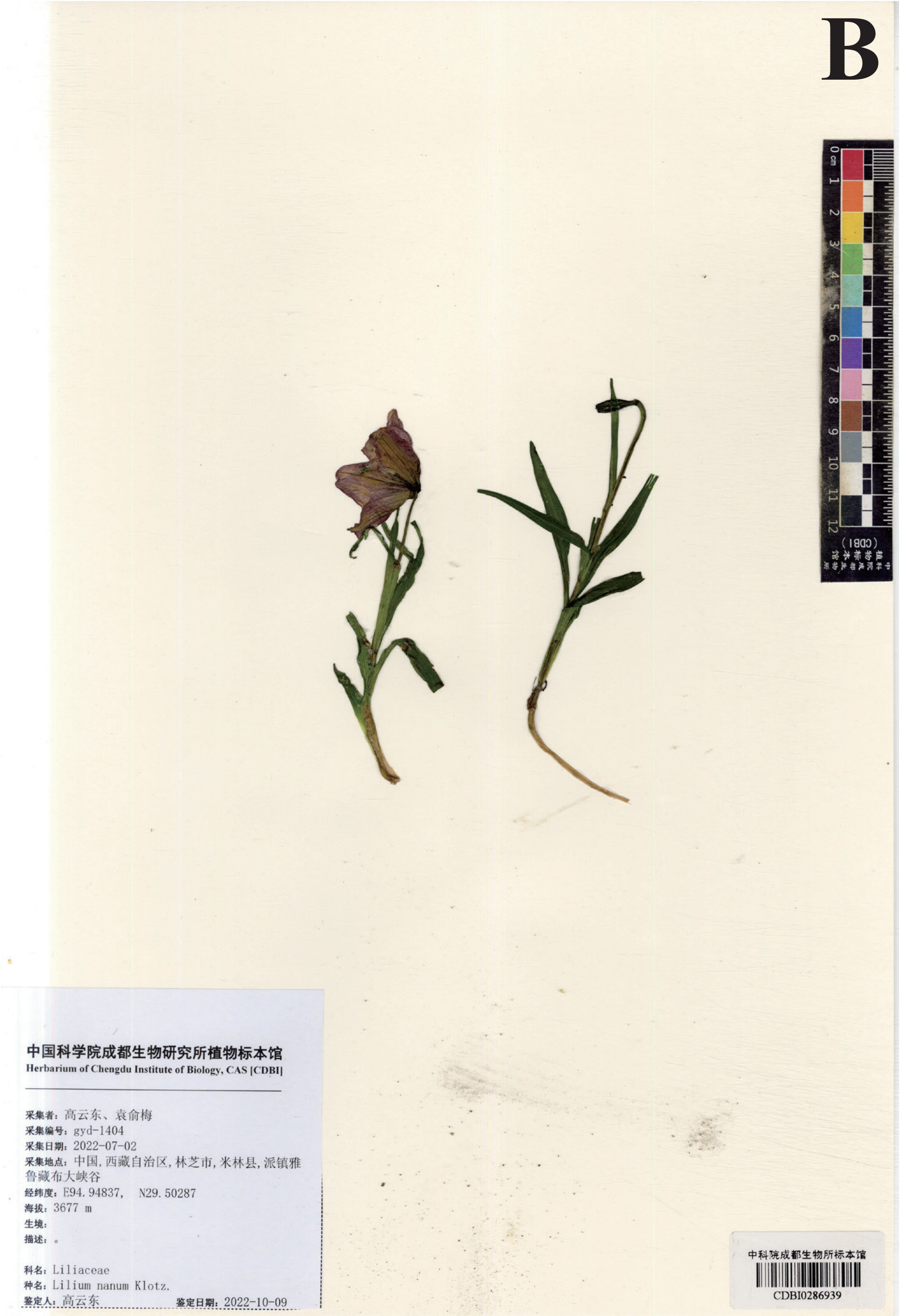
B
中国科学院成都生物研究所植物标本馆
Herbarium of Chengdu Institute of Biology, CAS [CDBI]
采集者：高云东、袁俞梅
采集编号：gyd-1404
采集日期：2022-07-02
采集地点：中国，西藏自治区，林芝市，米林县，派镇雅鲁藏布大峡谷
经纬度：E94.94837， N29.50287
海拔：3677 m
生境：
描述：。
科名：Liliaceae
种名：Lilium nanum Klotz.
鉴定人：高云东
鉴定日期：2022-10-09
中科院成都生物所标本馆
CDBI0286939
