# Supplementary material for: Lilium pseudonanum (Liliaceae), a Rare and Cryptic Species From Southeast Xizang, China
Source: Ecol Evol. 2025 Jul 10;15(7):e71738. doi: 10.1002/ece3.71738 (PMC12245480; doi:10.1002/ece3.71738)
Supplement: Supplementary file 1 — Appendix S1 [file ECE3-15-e71738-s001.zip › FigS2.pdf]

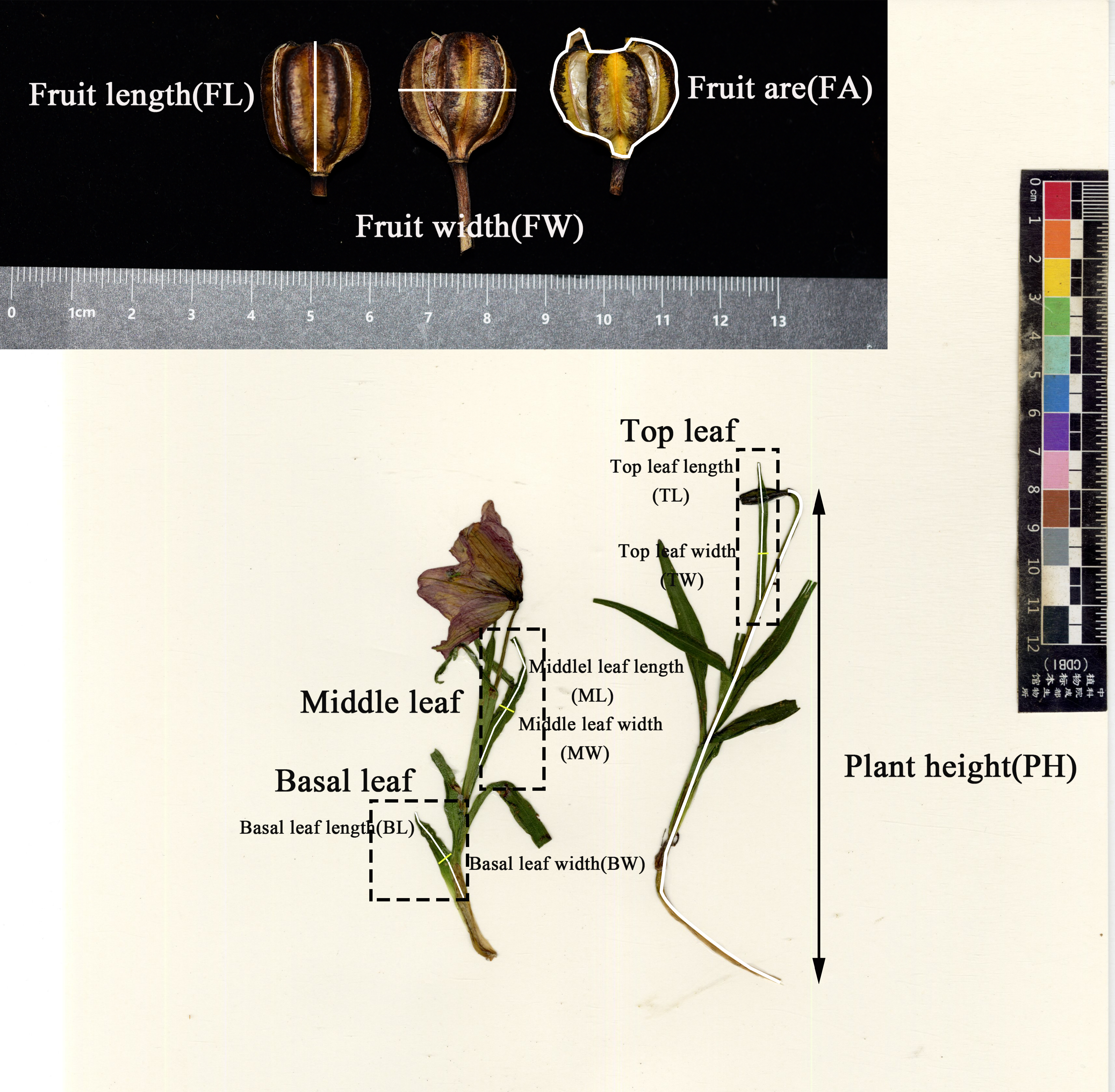
Fruit length(FL)
Fruit width(FW)
Fruit are(FA)
Top leaf
Top leaf length
(TL)
Top leaf width
(TW)
Middle leaf
Middlel leaf length
(ML)
Middle leaf width
(MW)
Basal leaf
Basal leaf length(BL)
Basal leaf width(BW)
Plant height(PH)
