# Supplementary material for: Lilium pseudonanum (Liliaceae), a Rare and Cryptic Species From Southeast Xizang, China
Source: Ecol Evol. 2025 Jul 10;15(7):e71738. doi: 10.1002/ece3.71738 (PMC12245480; doi:10.1002/ece3.71738)

Fig. S1 The holotype specimens of *L. nanum* and *L. pseudonanum*

*
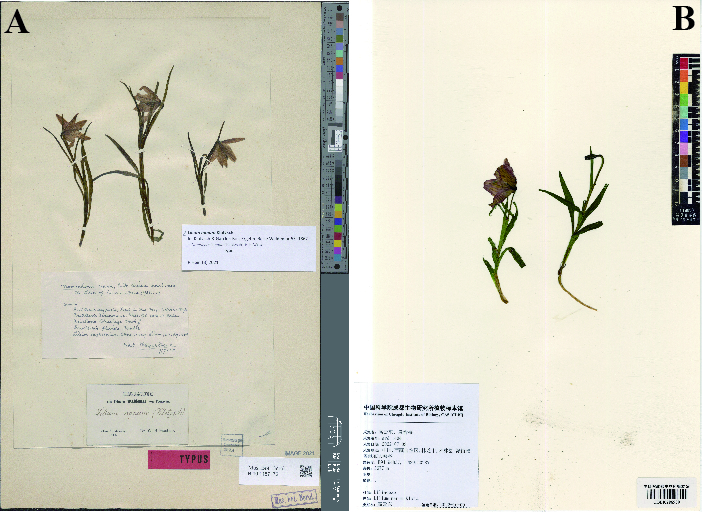
*

Note:A: The holotype of *Lilium nanum* (designated by Klotzsch in 1862, deposited at the B herbarium under accession number B 10 0307073); B: The holotype of *Lilium pseudonanum* (Y.D. Gao & Y.M. Yuan, deposited at the Chengdu Institute of Biology, Chinese Academy of Sciences, under holotype number CDBI0286939).

Fig. S2 Schematic for collecting quantitative data


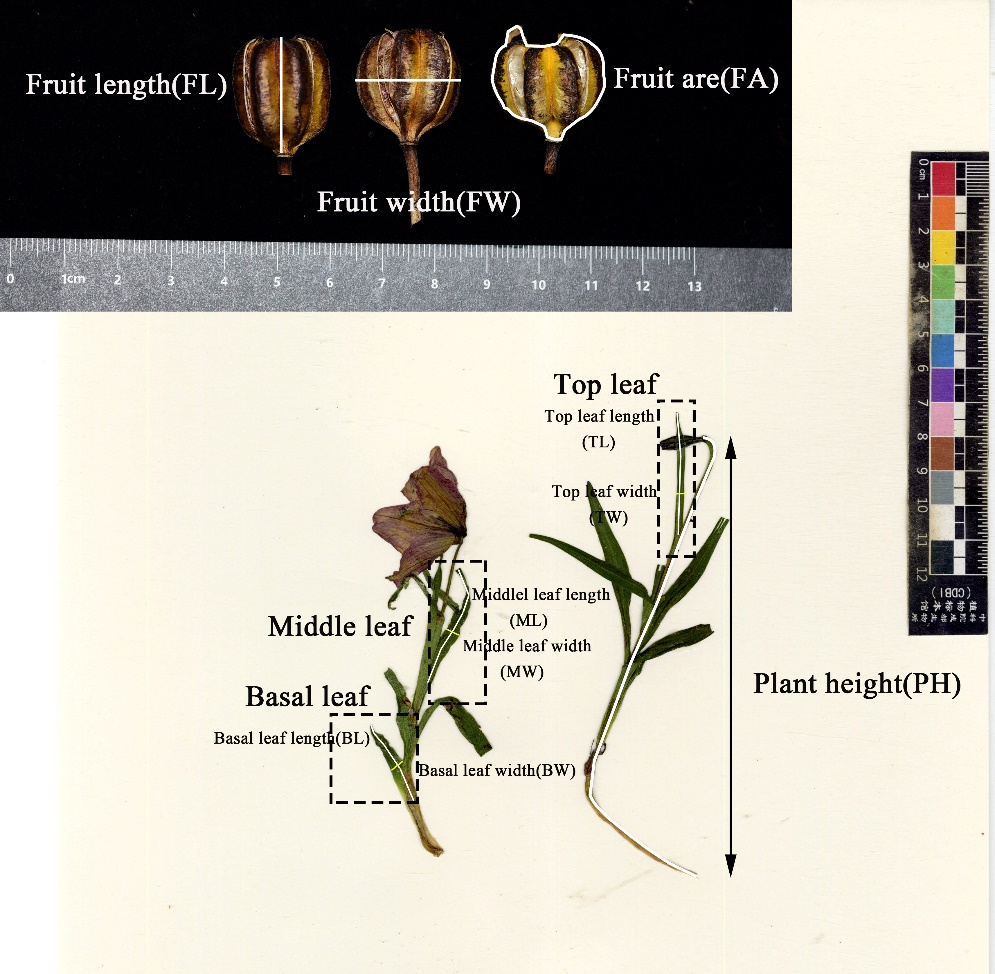


Fig. S3 Chloroplast CDS phylogenetic tree.


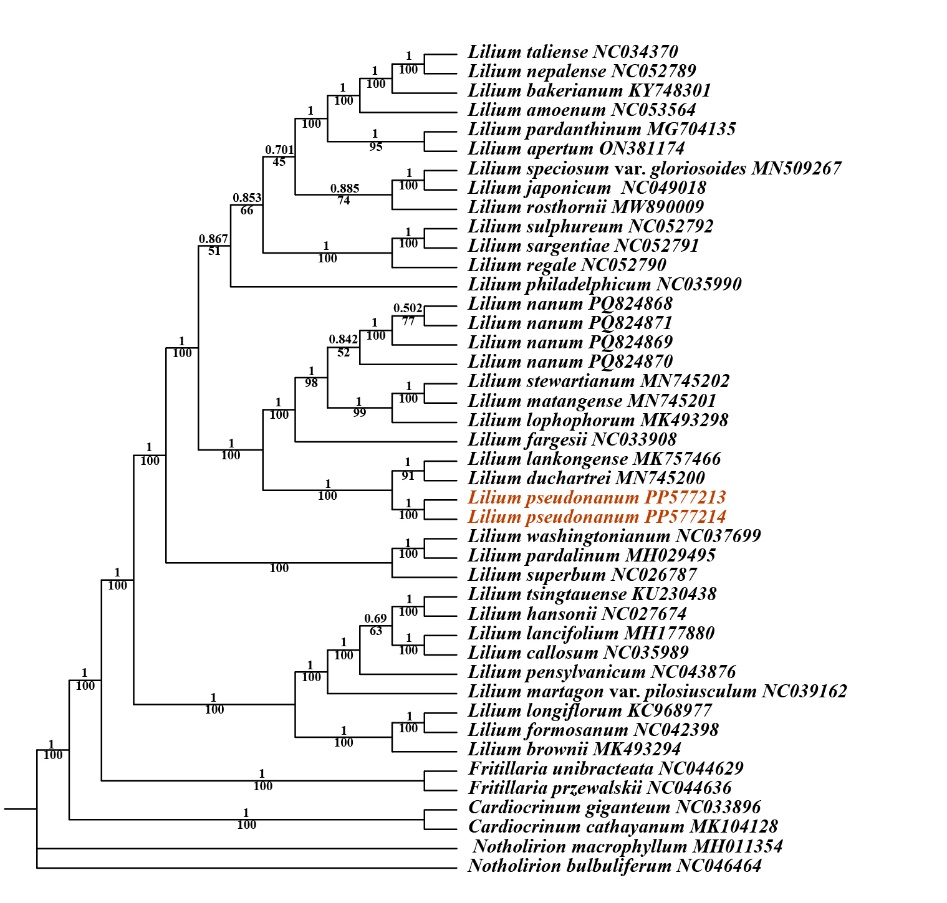


Fig. S4 Multi-label tree based on GRAMPA


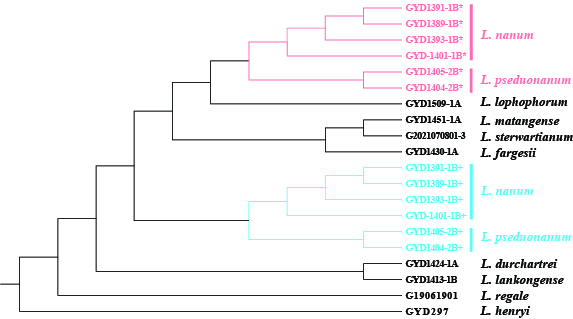


Fig. S5 Response curves of dominant environmental factors affecting the distribution of *Lilium nanum* (current ssp585)


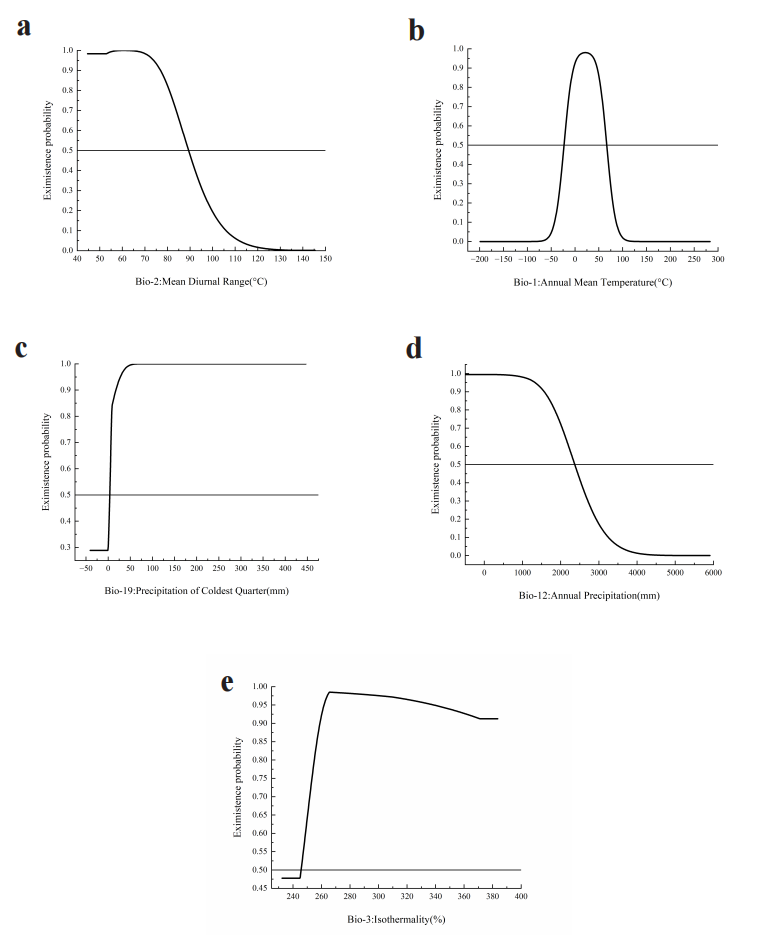

Supplement: Supplementary file 1 — Appendix S1 [file ECE3-15-e71738-s001.zip › Supplementary figures.docx]
