# Supplementary figures and images for: Lilium pseudonanum (Liliaceae), a Rare and Cryptic Species From Southeast Xizang, China
Source: Ecol Evol. 2025 Jul 10;15(7):e71738. doi: 10.1002/ece3.71738 (PMC12245480; doi:10.1002/ece3.71738)

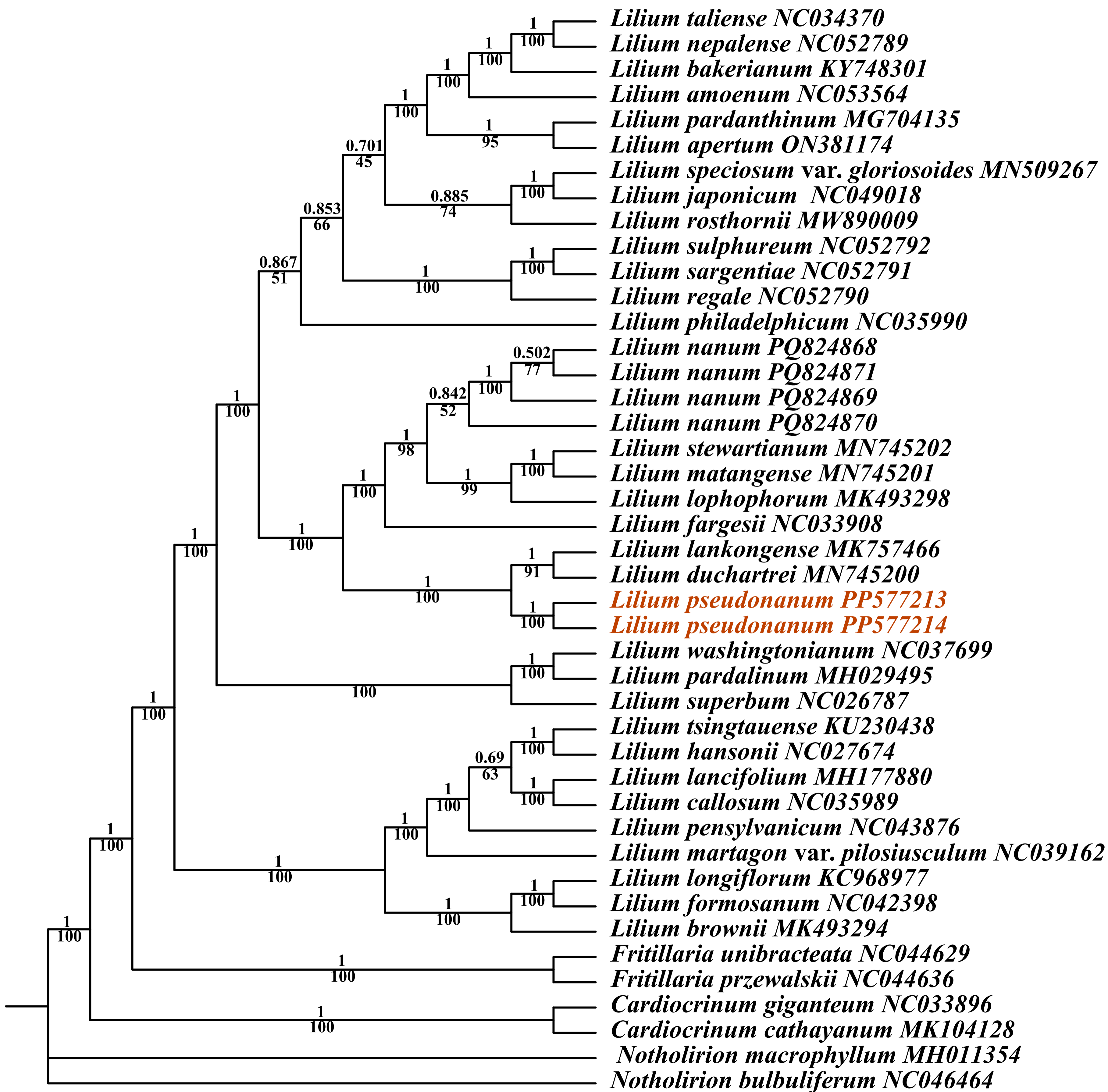

Supplement: Supplementary file 1 — Appendix S1 [file ECE3-15-e71738-s001.zip › FigS3.pdf]

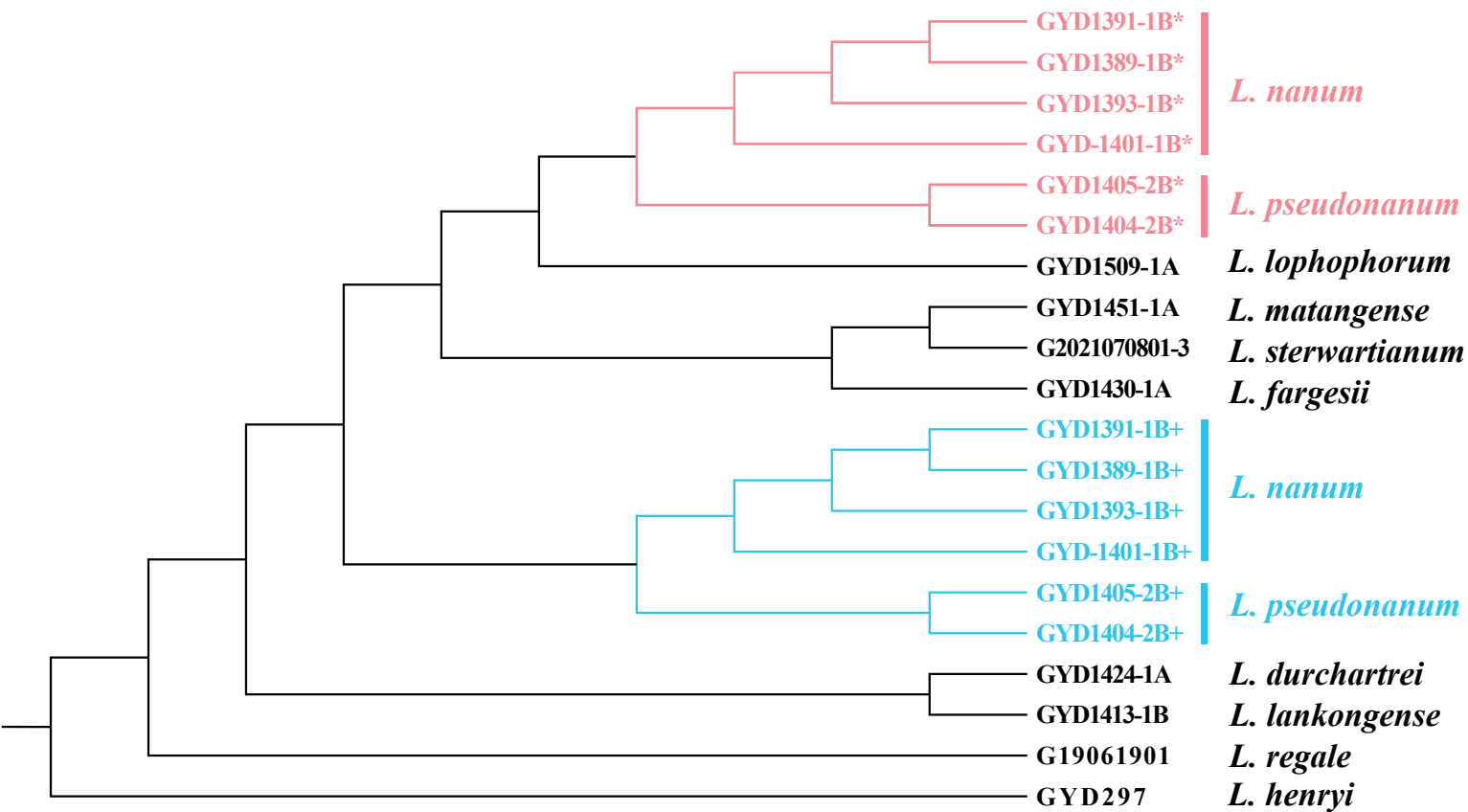

Supplement: Supplementary file 1 — Appendix S1 [file ECE3-15-e71738-s001.zip › FigS4.pdf]

**a**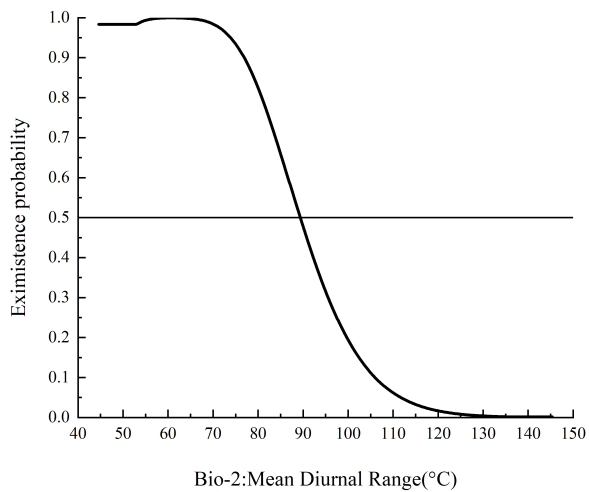**b**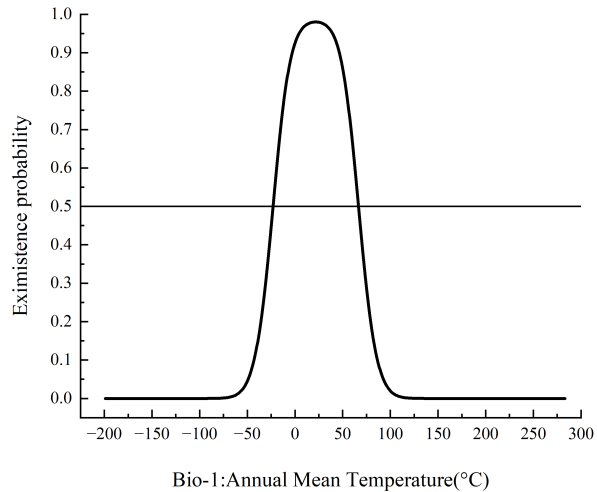**c**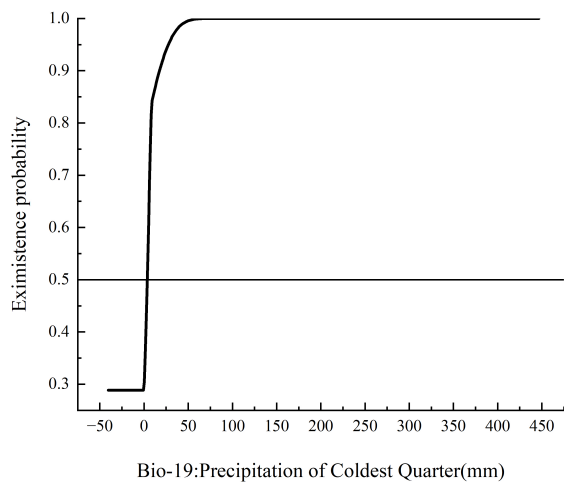**d**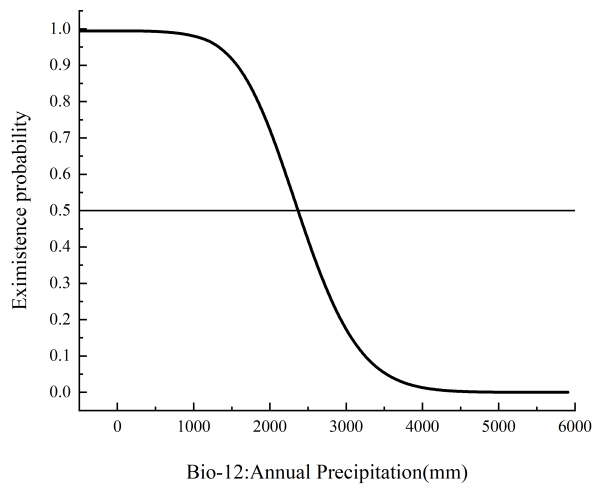**e**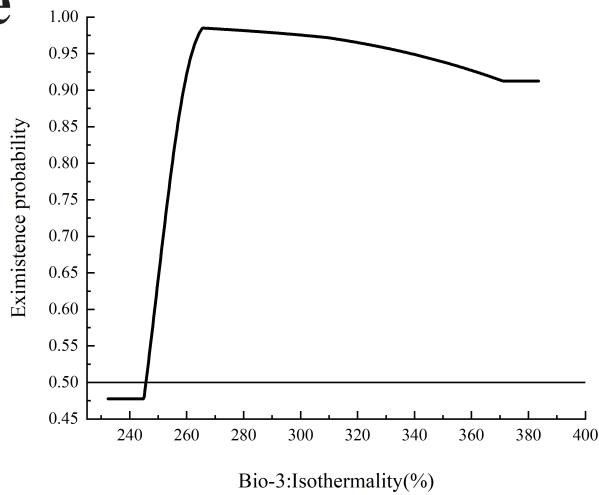

Supplement: Supplementary file 1 — Appendix S1 [file ECE3-15-e71738-s001.zip › FigS5.pdf]
